# The complete chloroplast genome of *Euphorbia ebracteolata* Hayata (Euphorbiaceae)

**DOI:** 10.1080/23802359.2020.1852904

**Published:** 2021-01-19

**Authors:** Mengli Wang, Xiufu Wan, Jiuwen Liang, Tan Li, Sheng Wang

**Affiliations:** State Key Laboratory Breeding Base of Dao-di Herbs, National Resources Center of Chinese Materia Medica, China Academy of Chinese Medical Sciences, Beijing, PR China

**Keywords:** **:***Euphorbia ebracteolata*, chloroplast genome, phylogenetic analysis

## Abstract

*Euphorbia ebracteolata* is a perennial medicinal plant and widely used in China for thousands of years. The complete chloroplast genome reported here is 163,090 bp in length, including two inverted repeats (IRs) of 26,699 bp, which are separated by a large single-copy (LSC) and a small single-copy (SSC) of 91,943 and 17,749 bp, respectively. The whole chloroplast genome of *E. ebracteolata* contains 112 genes, including 78 protein-coding genes, 30 transfer RNA, and 4 ribosome RNA. Phylogenetic analysis result strongly indicated that *E. ebracteolata* is closely related to *E. helioscopia.*

The genus Euphorbia is the largest in the spurge family, some of which have been used as medicinal plants for a long time. *Euphorbia ebracteolata* Hayata (Euphorbiaceae), a perennial herb distributed in the central and eastern of China. The root of this plant is poisonous, named ‘LangDu’ which is used as a traditional herbal medicine to treat edema, asthma, tuberculosis, and tumor diseases for thousands of years (Fu et al. 2006). Because ‘LangDu’ is widely used and extremely important, it is necessary to understand the background of the evolutionary biology of *E. ebracteolata*. Chloroplast genome is used as a source of valuable data for phylogenetic analysis, genetic diversity evaluation, and plant molecular identification (Dong et al. 2018; Sun et al. 2020). In this study, the completed chloroplast genome sequence of *E. ebracteolata* is determined which provides valuable genetic information for the phylogenetic studies.

The fresh samples of *E. ebracteolata* were collected from Huaining county Anhui province, China (30°44′1″N, 116°49′47″E). Voucher specimen was stored at the herbarium of Institute of Chinese Materia Medica (CMMI), China Academy of Chinese Medical Sciences with the specimen voucher number is 340822LY0035. Total genomic DNA from fresh leaves of a single individual using the method of Li et al. (2013). And the sequencing library was constructed and quantified following the methods introduced by Dong et al. (2017).

The whole-genome sequencing was conducted with 150 bp paired-end reads on the Illumina HiSeq X Ten platform. Contigs were assembled from the high-quality paired-end reads by using the SPAdes version 3.6.1 program (Kmer = 95) (Bankevich et al. 2012) after low-quality reads were filtered. The chloroplast genome contigs selected by the Blast program (Altschul et al. 1990), taken *Malva parviflora* (GenBank: MK860036) as the reference. The selected contigs were assembled using Sequencher version 4.10 (Gene Codes Corporation, Ann Arbor, MI http://www.genecodes.com). Gene annotation was performed with Plann (Daisie et al. 2015), taken the cp genome of *E. esula* (GenBank: KY000001) as the reference and manually corrected for codons and gene boundaries using BLAST searches. The annotated *E. ebracteolata* genomic sequence has been deposited into GenBank with the accession number MT830860.

The complete chloroplast genome reported here is 1,63,090 bp in length, including two inverted repeats (IRs) of 26,699 bp, which are separated by a large single-copy (LSC) and a small single-copy (SSC) of 91,943 and 18,089 bp, respectively. The overall GC-content of the chloroplast genome was 35.5%. The chloroplast DNA of *E. ebracteolata* comprised 112 distinct genes, including 78 protein-coding genes, 4 transfer RNA, and 30 ribosome RNA, do not contain *ycf15*. In these genes, 17 harbored a single intron, whereas two (ycf3 and clpP) contained double introns. The *rps12* gene is a trans-spliced gene with 5′ end located in the LSC region and the 3′ end located in the IR region. The gene *trnK*-*UUU* has the largest intron, which contains the *matK* gene.

To confirm the phylogenetic location of *E. ebracteolata* within the genus *Euphorbia*, total 11 complete cp genomes were downloaded from Genbank and the genus *Daphniphyllum* was taken as an outgroup. All chloroplast genome sequences were aligned using MAFFT (Katoh et al. 2019). Phylogenetic analysis was conducted based on IQ-tree using PhyloSuite under the TVM + F+I + G4 model with 1000 bootstrap replicates (Nguyen et al. 2015; Zhang et al. 2020). The phylogenetic tree showed that all species of *Eupho*rbia form a monophyletic group with 100% support, and *E. ebracteolata* was closely related to *E. helioscopia* (Figure 1). The chloroplast genome of *E. ebracteolata* provided a lot of genetic information for species conservation and taxonomy of genus *Eupho*rbia.

**Figure 1.**
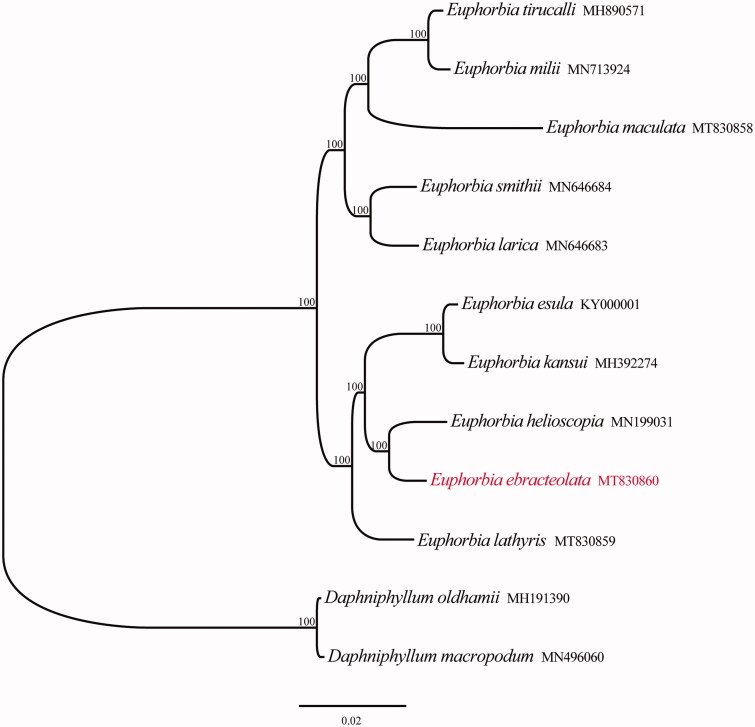
Phylogenetic tree reconstruction of 12 taxa using maximum likelihood (ML) methods in the chloroplast genome sequences. ML bootstrap support value presented at each node.

## Data Availability

The data that support the findings of this study are openly available in GenBank of NCBI https://www.ncbi.nlm.nih.gov/, reference number MT830860, raw data BioProject ID: PRJNA662166, Submission ID: SUB8183746.
